# A real-world analysis of 1,823 hospitalized osteoporotic fractures in Northeast China

**DOI:** 10.3389/fendo.2024.1520229

**Published:** 2025-01-07

**Authors:** Qi Meng, Xinwei Wang, Yuzhong Gao, William D. Leslie, Lisa M. Lix, Xianbao Shi, Bo Kan, Shuman Yang

**Affiliations:** ^1^ Department of Endocrinology, The First Affiliated Hospital of Jinzhou Medical University, Jinzhou, Liaoning, China; ^2^ Department of Epidemiology and Biostatistics, School of Public Health, Jilin University, Changchun, Jilin, China; ^3^ Department of Orthopedics, The First Affiliated Hospital of Jinzhou Medical University, Jinzhou, Liaoning, China; ^4^ Department of Internal Medicine, University of Manitoba, Winnipeg, MB, Canada; ^5^ Department of Community Health Sciences, University of Manitoba, Winnipeg, MB, Canada; ^6^ Department of Pharmacy, The First Affiliated Hospital of Jinzhou Medical University, Jinzhou, Liaoning, China; ^7^ Department of Clinical Laboratory, The Second Hospital of Jilin University, Changchun, Jilin, China

**Keywords:** osteoporotic fractures, epidemiology, real-world analysis, case series, aged people

## Abstract

**Context:**

There are limited real-world data evidence assessing the clinical characteristics of hospitalized osteoporotic fractures in China.

**Objective:**

To investigate the clinical characteristics of hospitalized major osteoporotic fractures in Northeast China.

**Methods:**

We identified hospitalized fracture patients aged 50 and over from the First Affiliated Hospital of Jinzhou Medical University between January 1, 2018, and December 31, 2022. Major osteoporotic fractures including hip, vertebral, forearm and wrist, and humerus were diagnosed based on x-ray reports extracted from the electronic medical records (EMR). The cause of fracture, comorbidities, surgical methods, and anti-osteoporotic medications (AM) use were extracted from EMR.

**Results:**

The study population included 1823 fracture patients, 510 males and 1313 females. Over half of fracture patients were aged over 70 years. Hip fractures accounted for 68.4% of all fractures in males and 57.9% in females. For those with hip fractures, the most common sites were the femoral neck (62.9%) and intertrochanteric (35.3%). Most fractures were due to falls (81.0% in males and 80.2% in females). The two most common comorbidities for males and females were hyperlipemia (45.9% vs. 47.1%) and hypertension (38.2% vs. 41.6%). Only 4.7% males and 8.6% females were treated with AM.

**Conclusions:**

Hip fractures, especially femoral neck fractures, accounted for the majority of osteoporotic fractures in a tertiary public hospital in Northeast China. Common comorbidities in these fracture patients were hyperlipemia and hypertension. There was a very low rate of AM use among these patients.

## Introduction

With an aging population, the prevalence of osteoporotic fracture was estimated to be 18.9% among elderly Chinese individuals (1). An investigation conducted in China found that the incidence rates of hip fractures per 100,000 in 2016 were 177.13 for females and 99.15 for males (2). Globally, there were 178 million new fractures and 25.8 million years lived with disability (YLDs) in 2019 (3). During 2005-2018, the one-year all-cause mortality after hip fracture in the UK and US were 28.3% and 27.7%, respectively (4). The direct cost for osteoporotic fracture treatment in Europe was approximately €36.3 billion in 2019 (5).

In China, several studies have been conducted on fracture incidence, prevalence, mortality, and associated risk factors (6–8), but there have been limited epidemiologic studies comprehensively investigating the sites, causes, comorbidities or treatment of osteoporotic fractures (8–11). For example, a national population-based study examined 254 fractures, which included 118 wrist fractures, 71 hip fractures and 65 spine fractures (8). However, this study did not consider comorbidities and treatment for osteoporotic fractures. One fracture study mainly examined outcomes and management after fracture (9), while another only examined hip fracture sites and causes (10). A retrospective study of 1539 elderly hip fracture patients found that 349 had one comorbidity and 785 had two or more comorbidities. These chronic comorbidities were not only associated with an increased risk of falls but also with poor prognosis (11).

The aim of this real-world study was to examine specific fracture sites, causes, comorbidities, and treatments of hospitalized incident major osteoporotic fractures in a tertiary public hospital in Northeast China.

## Methods

### Study setting and population

Data on major osteoporotic fractures were derived from the electronic medical records (EMR) of inpatient patients of the First Affiliated Hospital of Jinzhou Medical University from a five-year period between January 1, 2018 and December 31, 2022. The First Affiliated Hospital of Jinzhou Medical University a major tertiary public hospital (one of the highest-level hospitals in China) is located in Jinzhou, the center of western Liaoning. It provides health-care services for more than 1.29 million outpatients and 0.12 million inpatients each year. This study protocol was approved by the Institute of Research Board of the First Affiliated Hospital of Jinzhou Medical University (Approval number: KYLL202392).

Inclusion criteria included the following: (1) the cause of hip, forearm and wrist or humerus fracture was with low-energy trauma (generally falls from standing height and below), and vertebral fragility fractures that occurred during the study period and (2) Patients over 50 years of age with new fractures. The exclusion criteria were: (1) fractures due to high trauma (i.e., car accident and fall from above standing height), (2) pathological fractures (i.e., bone metastasis) or secondary osteoporotic fractures (i.e., rheumatoid arthritis, chronic nutritional deficiency, hyperthyroidism, chronic obstructive pulmonary disease, Parkinson’s disease, chronic liver disease, chronic kidney disease), (3) use of glucocorticosteroid medications, (4) periprosthetic femoral fracture, (5) the fracture occurred within half a year after a previous one, and (6) it was clearly described as “old hip fracture” by physicians in diagnosis text.

### Osteoporotic fracture diagnosis

This research considered major osteoporotic fractures, including hip, vertebral, forearm and wrist, and humerus; all fractures were diagnosed based on x-ray reports and extracted from the EMR. To ensure the validation of our fracture cases, we randomly called 30 fractures patients and confirmed all fracture cases. The hip fractures were classified into femoral neck, intertrochanteric femur, subtrochanteric femur and other unclassified sites (i.e., lesser trochanter, greater trochanter, femoral head). Clinical vertebral fractures were identified from T4 to L4. Forearm and wrist included radius (i.e., proximal radius, radius head), ulna (i.e., proximal ulna, ulna head), and carpal. Proximal humerus included greater tuberosity, lesser tuberosity, anatomical neck, surgical neck and humeral head. Because of the limited number of proximal humerus fractures, all proximal humerus factures were combined in the analyses without further classification. Multi-site fractures were defined as any two or more fractures of the hip, vertebrae, forearm and humerus at the same time. Prior fracture was defined as a history of any previous fracture occurred after 40 years old. Data on comminuted fractures were also collected.

### Other measures

We considered the following measures: patient age, year, season, hospitalization duration and the cause of fracture, comorbidities, surgical treatment of fractures, and anti-osteoporotic medications (AM) used in hospital post fracture; these data were extracted from EMR. In China, the seasons of spring, summer, autumn, and winter were from March to May, June to August, September to November, and December to February, respectively. The residence determined by checking the valid identity documents number. The causes of fracture included fall from standing height and below (the location of the fall was categorized into indoor, outdoor and unknown), sprain and other (e.g., low back pain and soreness with no apparent cause, after lifting heavy objects, bumps in the car or presented with discomfort in other parts of the body). Comorbidities assessed included type 2 diabetes mellitus (T2DM), hyperlipemia, hypertension, coronary heart disease (CHD), ischemic stroke, hemorrhagic stroke, respiratory diseases (including asthma, pneumonia, chronic bronchitis, emphysema and lung cancer), gastrointestinal diseases (including chronic gastritis, chronic enteritis, gastric cancer and colorectal cancer), which were ascertained from in-hospital diagnosis records and/or self-reported history. Hyperlipidemia was determined based on the blood lipid test results (including high-density lipoprotein cholesterol, low-density lipoprotein cholesterol, total cholesterol and triglycerides) (12). AM included bisphosphonates (i.e., zoledronic acid, alendronate sodium, ibandronat sodium and risedronate sodium), others (i.e., alfacalcidol, salmon calcitonin and menatetrenone) and the Chinese medicine.

### Statistical analysis

We descriptively analyzed the baseline characteristics, fracture sites, types, causes, comorbidities, and treatment by sex using frequencies, percentages, medians and inter-quartile ranges in males and females separately. Specially, we used the Chi-square test to compare the descriptive data including seasons, comorbidities (i.e., hyperlipemia, hypertension, ischemic stroke and respiratory diseases) between fracture sites. The *Fisher* exact probability method was used to compare residence between fracture sites. The Kruskal-Wallis test was used to test for differences of age, hospitalization duration, number of comorbidities between fracture sites. Test for trend of the incident fracture numbers from 2018 to 2022 by fracture site and sex was estimated from age and sex adjusted linear regression models. Two-sided *P* < 0.05 was considered to be statistically significant. All statistical analyses were performed using the SPSS software (version 24.0; SPSS, Chicago, IL).

## Results

### Participants and fractures

**Figure 1:**
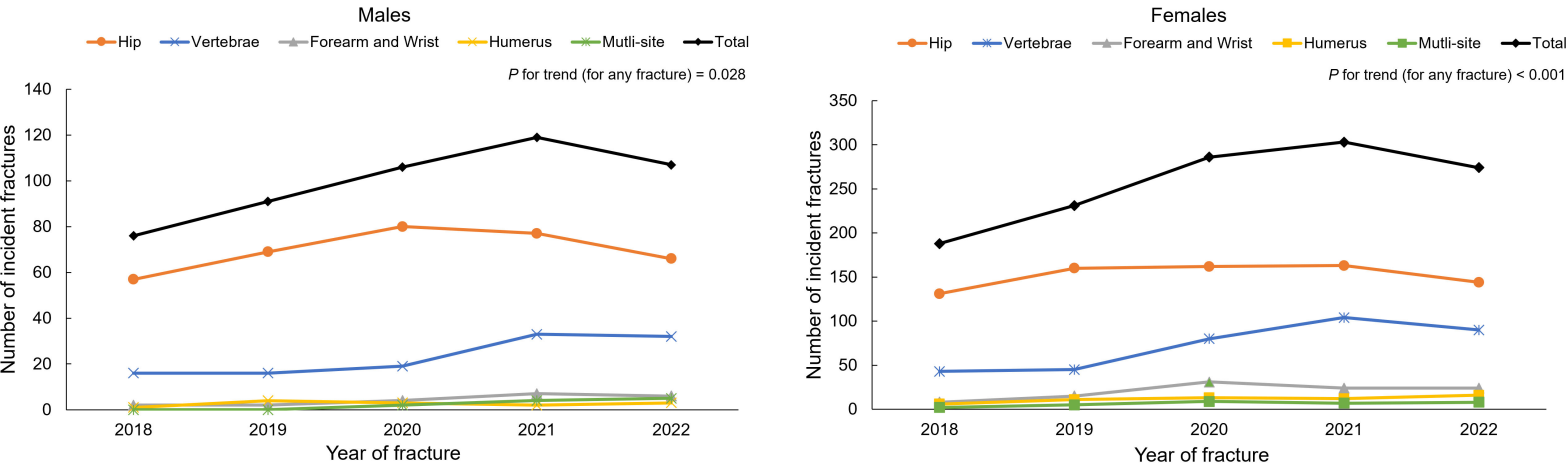
Numbers of first hospitalized fractures from 2018 to 2022 by skeletal site among males and females. *P* for trend was based on linear regression models by adjusting age.

We included a total of 1823 fracture patients, which involved 510 (28.0%) males and 1313 (82.0%) females (**Table 1**). Hip, vertebral, forearm and wrist, humerus and multi-site fractures accounted for 68.4%, 22.7%, 4.1%, 2.5% and 2.2% of all fractures in males and 57.9%, 27.6%, 7.8%, 4.4% and 2.4% in females, respectively. Among those fracture patients, 108 individuals had a prior fracture (26 in men and 80 in women, respectively). Prior fracture among hip fracture patients accounted for 4.6% in males and 7.1% in females. Overall, there was an increase in fracture numbers from 2018 to 2022 in males (*P* for trend for any fracture =0.028; **Figure 1**); females showed a similar trend (*P* for trend for any fracture < 0.001). Among those with hip fractures the most common sites involved were the femoral neck (62.9%; **Supplementary Table 1**) and intertrochanteric (35.3%). For vertebral fractures, lumbar and thoracic accounted for 64.0% and 49.8%, respectively. The most common site for forearm fracture was the distal radius (92.7%). Comparable results were noted for males and females. Multi-site fracture mainly comprised hip and forearm and wrist (38.1%; **Supplementary Table 2**), vertebral and forearm (28.6%), hip and vertebral (11.9%), and hip and humerus (11.9%). Among non-vertebral comminuted fractures, forearm and wrist and humerus were the most common types in males and females (**Supplementary Table 3**).

**Table 1:** Demographic characteristics of 1823 fracture patients by skeletal site and sex.

	All	Hip Only	Vertebrae Only	Forearm and Wrist Only	Humerus Only	Multi-site	*P*- between fracture sites^#^
Male							
N	510	349	116	21	13	11	
Age group, n (%)							<0.001^**^
50-59 years	71 (13.9)	39 (11.2) ^c^	19 (16.4) ^c^	8 (38.1)	4 (30.8)	1 (9.1)	
60-69 years	128 (25.1)	81 (23.2)	28 (24.1)	10 (47.6)	4 (30.8)	5 (45.5)	
70-79 years	129 (25.3)	94 (26.9)	30 (25.9)	2 (9.5)	0	3 (27.3)	
≥80 years	182 (35.7)	135 (38.7)	39 (33.6)	1 (4.8)	5 (38.5)	2 (18.2)	
Season of fracture, n (%)							0.414^*^
Spring and Winter	251 (49.2)	175 (50.1)	54 (46.6)	9 (42.9)	9 (69.2)	4 (36.4)	
Summer and Autumn	259 (50.8)	174 (49.9)	62 (53.4)	12 (57.1)	4 (30.8)	7 (63.6)	
Spring	129 (25.3)	78 (22.3)	37 (31.9)	5 (23.8)	6 (46.2)	3 (27.3)	
Summer	117 (22.9)	79 (22.6)	27 (23.3)	4 (19.0)	2 (15.4)	5 (45.5)	
Autumn	142 (27.8)	95 (27.2)	35 (30.2)	8 (38.1)	2 (15.4)	2 (18.2)	
Winter	122 (23.9)	97 (27.8)	17 (14.7)	4 (19.0)	3 (23.1)	1 (9.1)	
Hospitalization duration, (days)	7.0 (4.0,11.0)	8.0 (5.0,11.0) ^b^	5.0 (3.0,10.0)	7.0 (3.0,9.0)	7.0 (4.0,9.0)	7.0 (4.0,8.0)	<0.001^**^
Residence, n (%)							0.361^***^
Jinzhou City	444 (87.1)	309 (88.5)	96 (82.8)	18 (85.7)	11 (84.6)	10 (90.9)	
Non- Jinzhou City	66 (12.9)	40 (11.5)	20 (17.2)	3 (14.3)	2 (15.4)	1 (9.1)	
Prior fracture^##^, n (%)	26 (5.1)	16 (4.6)	7 (6.0)	0	1 (7.7)	2 (18.2)	0.187^***^
Female							
n	1313	760	362	102	58	31	
Age, n (%)							
50-59 years	131 (10)	51 (6.7) ^b,c,d^	32 (8.8) ^c^	36 (35.3) ^d^	10 (17.2)	2 (6.5)	<0.001^**^
60-69 years	328 (25.0)	146 (19.2)	126 (34.8)	37 (36.3)	13 (22.4)	6 (19.4)	
70-79 years	452 (34.4)	258 (33.9)	134 (37.0)	20 (19.6)	27 (46.6)	13 (41.9)	
≥80 years	402 (30.6)	305 (40.1)	70 (19.3)	9 (8.8)	8 (13.8)	10 (32.3)	
Season of fracture, n (%)							0.181^*^
Spring and Winter	613 (46.7)	359 (47.2)	178 (49.2)	42 (41.2)	21 (36.2)	13 (41.9)	
Summer and Autumn	700 (53.3)	401 (52.8)	184 (50.8)	60 (58.8)	37 (63.8)	18 (58.1)	
Spring	300 (22.8)	178 (23.4)	92 (25.4)	18 (17.6)	7 (12.1)	5 (16.1)	
Summer	332 (25.3)	198 (26.1)	81 (22.4)	24 (23.5)	16 (27.6)	13 (41.9)	
Autumn	368 (28.0)	203 (26.7)	103 (28.5)	36 (35.3)	21 (36.2)	5 (16.1)	
Winter	313 (23.8)	181 (23.8)	86 (23.8)	24 (23.5)	14 (24.1)	8 (25.8)	
Hospitalization duration, (days)	7.0 (4.0,10.0)	8.0 (6.0,12.0) ^b,c,d^	4.0 (3.0,7.0)	5.0 (3.0,8.0)	6.0 (3.0,8.0)	6.0 (4.0,10.0)	<0.001^**^
Residence, n (%)							0.207^***^
Jinzhou City	1176 (89.6)	690 (90.8)	321 (88.7)	86 (84.3)	52 (89.7)	27 (87.1)	
Non- Jinzhou City	137 (10.4)	70 (9.2)	41 (11.3)	16 (15.7)	6 (10.3)	4 (12.9)	
Prior fracture^##^, n (%)	80 (6.1)	54 (7.1)	20 (5.5)	3 (2.9)	0	3 (2.9)	0.064^***^

Hospitalization durations are presented as medians; ^*^Use the Chi-square test and comparison between binary classification of seasons; ^**^Use the Kruskal-Wallis test; ^***^Use the *Fisher* exact probability method.

^#^Excluding multiple fractures.

^##^Prior fracture was defined as a history of any previous fracture occurred after 40 years old.

^b^Compared to the vertebrae only group; ^c^Compared to the vertebrae only group; ^d^Compared to the vertebrae only group.

The overall number of fractures increased with age in males (*P* for trend = 0.009; **Table 1**). The majority of fractures occurred in autumn. The median length of hospitalization for any fractures was 7 days (interquartile range: 4.0-11.0 days in males and 4.0-10.0 in females). Hip fractures had the longest length of stay in males and females (median duration = 8 days in both sexes) and vertebral fractures had the shortest hospitalization duration (median duration = 5 days in males; median duration = 4 days in females).

### Causes of fractures

Overall, most fractures were due to falls (81.0% in males and 80.2% in females; **Table 2**). Falls occurred mainly indoors (73.4% in males and 81.7% in females). The proportion of vertebral fractures due to falls decreased to 46.6% in males and 51.4% in females; a more important role was played by other non-fall reasons such as no apparent cause, lifting heavy objects and bumps (50.9% in males and 43.1% in females).

**Table 2:** Causes of fractures by sex.

Sex/Cause of Fracture	All	Hip Only	Vertebrae Only	Forearm and Wrist Only	Humerus Only	Multi-site
Male (N=556)						
Fall, n (%)	413 (81.0)	318 (91.1)	54 (46.6)	18 (85.7)	12 (92.3)	11 (100.0)
Indoors, n (%)	303 (73.4)	243 (76.4)	42 (77.8)	4 (22.2)	5 (41.7)	9 (81.8)
Outdoors, n (%)	69 (16.7)	48 (15.1)	4 (7.4)	9 (50.0)	6 (50.0)	2 (18.2)
Unknown, n (%)	41 (9.9)	27 (8.5)	8 (14.8)	5 (27.8)	1 (8.3)	0
Sprain, n (%)	5 (1.0)	2 (0.6)	3 (2.6)	0	0	0
Others, n (%)	92 (18.0)	29 (8.3)	59 (50.9)	3 (14.3)	1 (7.7)	0
Female (N=1384)						
Fall, n (%)	1053 (80.2)	690 (90.8)	186 (51.4)	94 (92.2)	52 (89.7)	31 (100.0)
Indoors, n (%)	860 (81.7)	599 (74.7)	139 (74.7)	65 (69.2)	34 (65.4)	23 (74.2)
Outdoors, n (%)	116 (11.0)	59 (8.6)	18 (9.7)	21 (22.3)	12 (23.1)	6 (19.4)
Unknown, n (%)	77 (7.3)	32 (4.7)	29 (15.6)	8 (8.5)	6 (11.5)	2 (6.5)
Sprain, n (%)	26 (2.0)	3 (0.4)	20 (5.5)	1 (1.0)	2 (3.4)	0
Others, n (%)	234 (17.8)	67 (8.8)	156 (43.1)	7 (6.9)	4 (6.9)	0

Others include no apparent cause, lifting heavy objects, bump.

### Comorbidities of fractures

The three most frequent comorbidities for males overall were hyperlipemia (45.9%), hypertension (38.2%), and ischemic stroke (34.3%) (**Table 3**). Among females, the three most frequent comorbidities overall were hyperlipemia (47.1%), hypertension (41.6%) and T2DM (21.5%). For both sexes, hyperlipemia was the most frequent comorbidity in both single and multi-site fractures. Patients with one comorbidity were the most common, which included 31.0% of males and 34.9% of females. Three or more comorbidities were seen in 26.1% of males and 20.0% of females. For males, the prevalence of ischemic stroke and T2DM differed between fracture sites. Hypertension, T2DM, ischemic stroke and respiratory diseases showed significant differences between fracture sites in females.

**Table 3:** Comorbidities in fracture patients by sex.

Sex/Comorbidity	All	Hip Only	Vertebrae Only	Forearm and Wrist Only	Humerus Only	Multi-site	*P*-between fracture sites^#^
Male (N=556)							
Hyperlipidemia, n (%)	234(45.9)	159(45.6)	55(47.4)	7(33.3)	9(69.2)	4(36.4)	0.231
Hypertension, n (%)	195(38.2)	143(41.0)	38(32.8)	6(28.6)	5(38.5)	3(27.3)	0.335
Ischemic stroke, n (%)	175(34.3)	133(38.1) ^c^	36(31.0)	2(9.5)	2(15.4)	2(18.2)	0.015
Type 2 diabetes mellitus, n (%)	99(19.4)	78(22.3) ^b^	13(11.2)	2(9.5)	3(23.1)	3(27.3)	0.030^*^
Coronary heart disease, n (%)	67(13.1)	49(14.0)	14(12.1)	1(4.8)	3(23.1)	0	0.434^*^
Respiratory diseases, n (%)	57(11.2)	33(9.5)	19(16.4)	1(4.8)	1(7.7)	3(27.3)	0.168^*^
Hemorrhagic stroke, n (%)	44(8.6)	28(8.0)	12(10.3)	1(4.8)	0	3(27.3)	0.729^*^
Gastrointestinal diseases, n (%)	6(1.2)	5(1.4)	1(0.9)	0	0	0	–
Number of comorbidities, n (%)							0.008^**^
0	91(17.8)	63(18.1)	14(12.1)	10(47.6)	1(7.7)	3(27.3)	
1	158(31.0)	95(27.2)	50(43.1)	5(23.8)	5(38.5)	3(27.3)	
2	128(25.1)	89(25.5)	29(25.0)	5(23.8)	4(30.8)	1(9.1)	
≥3	133(26.1)	102(29.2)	23(19.8)	1(4.8)	3(23.1)	4(36.4)	
Female (N=1384)							
Hyperlipidemia, n (%)	618(47.1)	346(45.5)	184(50.8)	44(43.1)	29(50.0)	15(48.4)	0.307
Hypertension, n (%)	546(41.6)	340(44.7)	136(37.6)	34(33.3)	26(44.8)	10(32.3)	0.035
Type 2 diabetes mellitus, n (%)	282(21.5)	195(25.7)	56(15.5)	10(9.8)	16(27.6)	5(16.1)	<0.001
Ischemic stroke, n (%)	218(16.6)	150(19.7)	49(13.5)	12(11.8)	2(3.4)	5(16.1)	<0.001
Coronary heart disease, n (%)	164(12.5)	107(14.1)	40(11)	6(5.9)	7(12.1)	4(12.9)	0.093
Respiratory diseases, n (%)	82(6.2)	58(7.6)	21(5.8)	1(1.0)	1(1.7)	1(3.2)	0.025
Hemorrhagic stroke, n (%)	38(2.9)	28(3.7)	6(1.7)	1(1.0)	3(5.2)	0	0.098^*^
Gastrointestinal diseases, n (%)	32(2.4)	15(2.0)	14(3.9)	1(1.0)	2(3.4)	0	0.171^*^
Number of comorbidities, n (%)							<0.001^**^
0	273(20.8)	155(20.4)	69(19.1)	32(31.4)	11(19)	6(19.4)	
1	458(34.9)	233(30.7)	144(39.8)	43(42.2)	25(43.1)	13(41.9)	
2	319(24.3)	185(24.3)	100(27.6)	16(15.7)	8(13.8)	10(32.3)	
≥3	263(20.0)	187(24.6)	49(13.5)	11(10.8)	14(24.1)	2(6.5)	

Number of comorbidities include type 2 diabetes mellitus, hyperlipemia, hypertension, coronary heart disease, ischemic stroke, hemorrhagic stroke, respiratory diseases and gastrointestinal diseases.

^*^Use the Fisher exact probability method; ^**^Use the Kruskal-Wallis test; Others use Chi-square test.

^#^Excluding multiple fractures.

^b^Compared to the vertebrae only group; ^c^Compared to the vertebrae only group; ^d^Compared to the vertebrae only group.

### Surgeries and AM for fractures

Among hip fracture patients, 44.4% males and 56.2% females underwent replacement surgeries while 37.2% males and 28.2% females underwent internal fixation surgeries (**Table 4**). For vertebral fractures, the major treatment for patients was percutaneous kyphoplasty (PKP) (44.8% in males, 61.3% in females). Internal fixation was the major treatment for forearm, humerus and multi-site fractures in both sexes.

**Table 4:** Surgeries and anti-osteoporotic medication use in fracture patients by skeletal site and sex.

	All	Hip Only	Vertebrae Only	Forearm and Wrist Only	Humerus Only	Multi-site
Male (N=556)						
Surgeries, n (%)						
Internal fixation	173(33.9)	130(37.2)	9(7.8)	16(76.2)	9(69.2)	9(81.8)
External fixation	0	0	0	0	0	0
Total hip arthroplasty or hemiarthroplasty/artificial femoral head replacement/humeral head replacement/shoulder replacement/radial head replacement	156(30.6)	155(44.4)	NA	0	0	1(9.1)
Percutaneous kyphoplasty	52(10.2)	NA	52(44.8)	NA	NA	0
Percutaneous vertebroplasty	7(1.3)	NA	6(4.3)	NA	NA	1(9.1)
Conservative treatment	129(25.3)	66(18.9)	49(42.5)	5(23.8)	4(30.8)	2(18.2)
Anti-osteoporotic medications, n (%)						
Zoledronic acid	21(4.1)	6(1.7)	15(12.9)	0	0	0
Chinese medicine	3(0.6)	2(0.6)	1(0.9)	0	0	0
Female (N=1384)						
Surgeries, n (%)						
Internal fixation	371(28.3)	214(28.2)	19(5.2)	81(79.4)	43(74.1)	14(45.2)
External fixation	1(0.1)	0	0	1(1.0)	0	0
Total hip arthroplasty or hemiarthroplasty/artificial femoral head replacement/humeral head replacement/shoulder replacement/radial head replacement	439(33.4)	427(56.2)	NA	0	3(5.2)	9(29.0)
Percutaneous kyphoplasty	222(16.9)	NA	222(61.3)	NA	NA	0
Percutaneous vertebroplasty	32(2.4)	NA	26(7.2)	NA	NA	6(19.4)
Conservative treatment	253(18.3)	122(16.1)	95(26.2)	20(19.6)	12(20.7)	4 (12.9)
Anti-osteoporotic medications, n (%)						
Bisphosphonates	106(8.1)	15(2.0)	87(24.0)	0	1(1.7)	3(9.7)
Zoledronic acid	102(7.8)	15(2.0)	84(23.2)	0	1(1.7)	2(6.5)
Ibandronate sodium	3(0.2)	0	2(0.6)	0	0	1(3.2)
Risedronate sodium	1(0.1)	0	1(0.3)	0	0	0
Others ^*^	4(0.3)	0	4(1.1)	0	0	0
Chinese medicine	3(0.2)	1(0.1)	2(0.6)	0	0	0

^*^Others included alfacalcidol, salmon calcitonin and menatetrenone.

The major AM in fracture patients was zoledronic acids in males and females (**Table 4**). Hospitalized vertebral fractures had the highest proportion of AM use (13.8% in males, 25.7% in females).

## Discussion

In this five-year retrospective study, we found that hip fractures predominated in hospitalized osteoporotic fractures, especially for femoral neck and intertrochanteric fractures. Hip and forearm and wrist were the most common combination among multi-site fractures. Most of the fractures were due to falls. Hyperlipidemia and hypertension were the most frequent comorbidities in both males and females. The most common surgical treatment for hip fracture was replacement surgery, forearm and humerus fractures internal fixation, vertebral fractures PKP. AM were not commonly used for fracture patients during hospitalization.

In our study, hip fractures accounted for the largest proportion of hospitalized fractures. This is consistent with a previous Chinese study (13), which found that hip fractures accounted for the highest percentage of 43.67%. This may be due to the fact that hip fractures typically undergo surgical treatment. Although vertebral fractures are the most common type of osteoporotic fractures in the elderly, the majority of vertebral fractures are clinically silent. Vertebral fractures without back pain will not usually present to hospital, so most vertebral fractures remain largely undiagnosed. Thus, only about one-quarter to one-third of vertebral fragility fractures are observed in the hospital setting (14). Wang et al. investigated the epidemiology of hip fracture in Hefei, China, and found that femoral neck fracture was the most common subtype for males (53.3%) and females (61.7%) (10). A US study obtained similar results when hip fracture, unspecified and transcervical fractures were combined (15). Our study also found that femoral neck was the most common type of hip. Research related to multiple sites of fractures is still scarce. In our study, we found that hip and forearm fractures were the most common combination; this is likely due to falls.

During hot days, dehydration, orthostatic hypotension and an increased fluid intake which leads to frequent urination may contribute to increased risk of summertime falls and fall-related fractures in the elderly (16). Feng et al. (17) conducted a research in 198 cities of China, and found that an increased risk for hip fractures in adults was associated with weather conditions, with the relative risk being 1.079 (95% confidence interval [CI], 1.074-1.083) for precipitation and 1.558 (95% CI, 1.546-1.570) for greater temperature. Our study also found that fractures were more likely to occur in summer and autumn. In the present research, the average duration of hospitalization for hip fracture patients was 8 days, which had the longest average hospital stay compared to other fractures. This was shorter than an Italian study, which demonstrated that the average length of stay for hip fracture surgery patients was 10.8 days in the orthopedic department (18). This is likely due to the new Diagnosis-related Groups (DRG) pricing and payment policy in China (19).

Our findings that hyperlipidemia and hypertension were the most common comorbidities were supported by previous studies. For example, Jin et al. found that the prevalence of hyperlipidemia in north-eastern China was highest at more than 50% (20). Wei et al. suggested that the prevalence of hypertension in hip fracture was 45.02% (21). In addition, Jiang et al. found that hypertension was present in 51.8% of hip fracture patients (22). The proportion of patients with 2+ comorbidities in this study is consistent with the findings of a Danish study, in which more than 40% of fracture patients exhibited 2+ comorbidities at the time of fracture (23). A study of chronic diseases in Beijing, China, showed that the number of community- based older adults with ≥3 chronic conditions increased significantly from 13.4% in 2004 to 30.5% in 2017 (24). Because comorbidities cause excessive mortality for fracture patients (23, 25), current osteoporosis management guidelines may need to pay attention to chronic conditions such as hyperlipidemia and hypertension following a fracture (26).

In our study, the use of AM among osteoporotic fracture patients was very low. A similar situation has been noted in South Korea (27) and several Asian countries (28). The use of AM in the Chinese mainland among postmenopausal women in the 6 months after a fragility hip fracture was only 6.5% (28). Some reasons are likely to cause low prescription of AM. First, Orthopedic surgeons commonly prioritize the surgery procedures for fracture treatments, and AM treatment may be overlooked. Second, fracture patients in China lack knowledge about AM (29). Better public education on osteoporosis and AM in the elderly in China is warranted.

Fracture registries have been established in several countries such as Spain, the UK, Australia and New Zealand. The fracture registries had the potential to expedite surgery and improve clinical care of fracture patients over time (30, 31). However, such high-quality fracture management program is not available in China. There are still multiple barriers for established this program; they include the complexity of program implementation, insufficient international collaboration and incentives, the absence of national guideline support and lack of digital health applications for communication between health providers, insufficient number of health providers and beds, and poor understanding about the effectiveness of this program (32). However, some research works in China had been done. For example, a recommended core variable set has been selected for the establishment of a Chinese national hip fracture registry; such measure is proposed to examine and improve the care of over half a million people each year (33). The co-managed care model, considered the clinical and financial acceptability, and the priority for resource allocation for osteoporosis management, was developed; this is proved to be highly cost-effective for hip fracture patients’ management in China (34). Lastly, our and other studies (8, 10, 11) provided baseline data for major osteoporotic fracture treatment and management status in China.

Several limitations are acknowledged. First, this study only included clinical vertebral fractures, not radiological vertebral fractures. Second, this study was conducted in a tertiary class-A public hospital in Jinzhou, China, which has inherent limitations in epidemiological and clinical characteristics. The findings may not be able to generalize to other hospitals or regions. Third, this study lacked information on post-hospitalization outcomes which would help to better understand the effectiveness of different treatment options, including factors influencing prognosis and the incidence of secondary fractures.

## Conclusion

This study found that hip fractures, especially those involving the femoral neck, were the most common hospitalized osteoporotic fracture in a public hospital in Northeast China. Thus, hip fracture remains a major problem for orthopedic clinicians. Most fractures were caused by falls and greater attention to falls prevention may substantially reduce the risk of fractures. Comorbidities, such as hyperlipidemia and hypertension, are common in the elderly population with fractures. These findings may help to refine existing guidelines for fracture diagnosis, treatment and prevention.

## Data Availability

The raw data supporting the conclusions of this article will be made available by the authors, without undue reservation.

## Supplementary material

Supplementary Table 1 Fractures subtypes for hip, vertebrae, forearm and wrist, and humerus by sex.

Supplementary Table 2 Multi-site fracture data by sex.

Supplementary Table 3 Comminuted fractures for hip, forearm and wrist, and humerus by sex.
