# Supplementary material for: A real-world analysis of 1,823 hospitalized osteoporotic fractures in Northeast China
Source: Front Endocrinol (Lausanne). 2025 Jan 7;15:1520229. doi: 10.3389/fendo.2024.1520229 (PMC11746098; doi:10.3389/fendo.2024.1520229)
Supplement: Supplementary Table 1 — Fractures subtypes for hip, vertebrae, forearm and wrist, and humerus by sex. [file Table1.docx]

**Supplementary Table 1. Fractures subtypes for hip, vertebrae, forearm and wrist, and humerus by sex**

| **Fracture Site** | **Hip Only, n (%)** | | | | **Vertebrae Only,**  **n (%)** | | **Forearm and Wrist Only,**  **n (%)** | | | **Humerus Only,**  **n (%)** |
| --- | --- | --- | --- | --- | --- | --- | --- | --- | --- | --- |
|  | Femoral neck | Intertrochanteric | Subtrochanteric | Unclassified proximal femur | Thoracic vertebra | Lumbar vertebra | Distal radius | Distal ulna | Carpal | Proximal humerus |
| Male  (N=499) | 201(57.6) | 144(41.3) | 7(2.0) | 7(2.0) | 51(44.0) | 82(70.7) | 17(81.0) | 6(28.6) | 4(19.0) | 13(100.0) |
| Female  (N=1282) | 497(65.4) | 247(32.5) | 11(1.4) | 12(1.6) | 187(51.7) | 224(61.9) | 97(95.1) | 47(46.1) | 5(4.9) | 58(100.0) |
| All  (N=1781) | 698(62.9) | 391(35.3) | 18(1.6) | 19(1.7) | 238(49.8) | 306(64.0) | 114(92.7) | 53(43.1) | 9(7.3) | 71(100.0) |

All humerus fractures were proximal humerus fractures.

**Supplementary Table 2.** **Multi-site fracture data by sex**

|  | **Total** | **Hip &**  **Forearm and Wrist** | **Vertebrae & Forearm and Wrist** | **Hip &**  **Humerus** | **Hip &**  **Vertebrae** | **Vertebrae & Humerus** | **Forearm and Wrist & Humerus** |
| --- | --- | --- | --- | --- | --- | --- | --- |
| Male, n (%) | 11(26.2) | 2(18.2) | 3(27.3) | 3(27.3) | 2(18.2) | 1(9.1) | 0 |
| Female, n (%) | 31(73.8) | 14(45.2) | 9(29.0) | 2(6.5) | 3(9.7) | 1(3.2) | 2(6.5) |
| All, n (%) | 42(100.0) | 16(38.1) | 12(28.6) | 5(11.9) | 5(11.9) | 2(4.8) | 2(4.8) |

Bolding indicates the most prevalent fracture combination.

**Supplementary Table 3. Comminuted fractures for hip, forearm and wrist, and humerus by sex**

| **Comminuted fracture site** | **Hip** | **Forearm and Wrist** | **Humerus** |
| --- | --- | --- | --- |
| Male, n (%) | 28(8.0) | 6(28.6) | 4(30.8) |
| Female, n (%) | 32(4.2) | 25(24.5) | 11(19.0) |
